# Utilisation of outpatient medical services in Germany – Results from GEDA 2019/2020-EHIS

**DOI:** 10.25646/8555

**Published:** 2021-09-15

**Authors:** Franziska Prütz, Alexander Rommel, Julia Thom, Yong Du, Giselle Sarganas, Anne Starker

**Affiliations:** Robert Koch Institute, Berlin Department of Epidemiology and Health Monitoring

**Keywords:** OUTPATIENT CARE, CANCER SCREENING, PSYCHOTHERAPY, BLOOD PRESSURE MONITORING, MEDICATION

## Abstract

Outpatient health care provision plays an important role in the identification and treatment of health problems. Data are needed on the utilisation of health care services and their determinants to enable health policy decision-making and needs-based care provision. The analyses set out in this article are based on current data on the utilisation of outpatient health care services. The data stem from the German Health Update (GEDA 2019/2020-EHIS), a nationwide cross-sectional survey of the resident population in Germany that is undertaken as part of the health monitoring conducted at the Robert Koch Institute.

Around 80% of the population aged 18 or over were treated at least once within twelve months by a general practitioner, 60% by a specialist, and 10% received psychiatric or psychotherapeutic treatment. Less than half of those eligible had had a stool test during the past two years, and just over half had had a colonoscopy in the past ten years. Around 80% of women and 70% of men had had their blood pressure checked within the last year, and 60% had had their blood cholesterol or blood sugar levels monitored. Over 50% reported that they had taken medically prescribed drugs in the past two weeks. In general, most of the indicators under study suggest that utilisation increases with age and that utilisation is higher among women than men, with the exception of psychiatric and psychotherapeutic services, among others.

## 1. Introduction

Outpatient health care plays an important role in identifying and treating health problems. The largest area is outpatient medical care and psychotherapy. In Germany, these services are mainly provided by office-based physicians and psychotherapists. As they are generally the first point of contact in the health care system, they determine the need for and provide treatment, carry out examinations, and, if necessary, arrange for the provision of further health care and social services [1]. Around 90% of adults in Germany utilise outpatient medical or psychotherapeutic services every year [2].

Medical care also includes blood pressure monitoring, and cholesterol and blood sugar tests. These tests play a key role in the prevention, diagnosis and management of cardiovascular diseases and diabetes, and are important aspects of quality of care. Health surveys have identified a significant increase in the number of blood pressure checkups conducted among people with high blood pressure [3] and a decrease in undetected high blood pressure [4] and unknown diabetes [5] in Germany between 1997/1998 and 2008 to 2011.

**GEDA 2019/2020-EHIS**

Fifth follow-up survey of the German Health Update

**Data holder:** Robert Koch Institute

**Objectives:** Provision of reliable information on the health status, health behaviour and health care of the population living in Germany, with the possibility of European comparisons

**Study design:** Cross-sectional telephone survey

**Population:** German-speaking population aged 15 and older living in private households that can be reached via landline or mobile phone

**Sampling:** Random sample of landline and mobile telephone numbers (dual-frame method) from the ADM sampling system (Arbeitskreis Deutscher Markt- und Sozialforschungsinstitute e.V.)

**Sample size:** 23,001 respondents

**Study period:** April 2019 to September 2020

**GEDA survey waves:**

▶ GEDA 2009
▶ GEDA 2010
▶ GEDA 2012
▶ GEDA 2014/2015-EHIS
▶ GEDA 2019/2020-EHIS

Further information in German is available at www.geda-studie.de

Medication is also an essential aspect of the treatment of health impairments, disorders, and diseases. Between 2014 and 2015, more than half of the population took medication prescribed by a doctor within a two-week period; among people 65 or above it was over 85% [6].

Preventive care, which includes vaccinations and cancer screening, also falls under the responsibility of outpatient health care. As such, preventive care also includes colorectal cancer screening, which is offered to people with statutory health insurance aged 50 or above in the form of stool tests and colonoscopies at different intervals depending on their age and sex. The costs are covered by statutory health insurers, and utilisation is voluntary [7]. Organised colorectal cancer screening was established in July 2019 in Germany and it involves inviting patients to screening and providing them with information about the screening. Previously, claims data from statutory health insurers in Germany demonstrated that around 18% of those eligible had undertaken a stool test for hidden blood (2017–2018) and that around 15% had undergone a colonoscopy (2009– 2018) [8]. It should be noted that in addition to colorectal cancer screening, colonoscopies are also used to determine the cause of symptoms, which leads to its higher overall utilisation [9].

Andersen’s Behavioural Model of Health Services Use describes a number of factors that influence the utilisation of health services [10, 11]. Andersen distinguishes between three groups of factors: (i) predisposing factors such as sex, age, education and professional status, (ii) enabling factors, e.g. income, types of health insurance and the accessibility of facilities, and (iii) need factors, of which a person’s health plays a central role [10]. If predisposing or enabling factors have a strong impact on utilisation that cannot be explained by different medical needs can result in the development of social inequalities in health care provision.

In order to develop health policy and ensure needs-based care provision, including the avoidance of overuse, underuse and misuse, information is required about the utilisation of health care services and their determinants [12]. For example, people with depressive symptoms seek help much more often in regions with a relatively large number of psychotherapists [13]. Analyses of the use of outpatient care can be carried out using claims data from health insurers and associations of statutory health insurance physicians as well as with data from population-based surveys. Survey data on utilisation are available, among others, from the health monitoring at the Robert Koch Institute. In contrast to claims data, survey data enable a more differentiated description of social and other determinants [14–16].

This article is based on key data from the study GEDA 2019/2020-EHIS on the current utilisation of general and specialist medical services, including psychiatric and psychotherapeutic care, by adults in Germany. It sets out results on the utilisation of selected outpatient services: stool test, colonoscopy, measurement of blood pressure, blood cholesterol and blood sugar by health professionals, and the utilisation of medically prescribed drugs. With regard to the factors influencing the utilisation of outpatient services, we focus on the predisposing factors of age, gender and education.

## 2. Methodology

### 2.1 Study design and sample

The German Health Update (GEDA) is a nationwide cross-sectional survey of the resident population in Germany. The GEDA study has been conducted by the Robert Koch Institute (RKI) on behalf of the German Federal Ministry of Health at multi-year intervals since 2008 and is part of the health monitoring at the RKI [17, 18]. The fifth follow-up survey, GEDA 2019/2020-EHIS, took place between April 2019 and September 2020. As in the 2014/2015 wave, the questionnaire of the European Health Interview Survey (EHIS) was fully integrated [19, 20]. GEDA 2019/2020-EHIS was conducted as a telephone interview survey using a computer assisted, fully structured interview (i.e. Computer Assisted Telephone Interview, CATI). It was based on a random sample of landline and mobile telephone numbers (dual-frame method) [21]. The sample comprised the population aged 15 years and older living in private households and with permanent residency in Germany. A total of 23,001 people provided complete interviews for the GEDA 2019/2020-EHIS study. Based on the standards of the American Association for Public Opinion Research (AAPOR), the response rate was 21.6% (RR3) [22]. A detailed description of the methodology used for GEDA 2019/2020-EHIS, including an explanation and differentiated presentation of the response rates, can be found in Allen et al. in this issue of the Journal of Health Monitoring [23].

### 2.2 Indicators

#### Utilisation of medical services

Data on the utilisation of medical services were collected using the question: ‘When was the last time you consulted a GP (general practitioner) or family doctor on your own behalf?’. The question on the utilisation of specialist services used a similar wording asking for consultations with medical or surgical specialists. Two dichotomous variables were formed to differentiate between respondents who had seen a GP, as well as those who had consulted a specialist in the last twelve months, from respondents who had not sought the corresponding medical care during this period.

#### Utilisation of psychiatric and psychotherapeutic services

Data on the utilisation of specialist mental health services were specifically recorded for psychological complaints and mental disorders. The participants were asked: ‘In the past twelve months have you visited on your own behalf a psychologist, psychotherapist or psychiatrist for counseling, examination or treatment?’. The possible responses were ‘Yes’,‘No’, ‘Don’t know’ and ‘Prefer not to answer’. When ‘psychotherapeutic and psychiatric services’ are referred to in the following, they also include services provided by psychologists without a licence to practice medicine, such as those provided in the context of outpatient addiction counselling.

#### Utilisation of stool test and colonoscopy

GEDA 2019/2020-EHIS collected data on colorectal cancer screening using the following questions: ‘When was the last time you had a test for hidden blood in your stool?’ and ‘When was the last time you had a colonoscopy?’. The possible responses were periods ranging from ‘Within the last twelve months’ to ‘Ten years ago or longer’. The respondents could also answer ‘Never’. The resulting data can be used to assess whether the last examination took place in accordance with the guidelines for colon cancer screening [7]. The analyses are based on routine stool tests and colonoscopies. This means a stool test within the last twelve months for women and men aged between 50 and 54; a stool test within the last two years for women and men aged 55 or over; and a colonoscopy within the last ten years for men aged 50 or above, and for women aged 55 or above.

#### Blood pressure, blood cholesterol and blood sugar measurement by health professionals

Data was collected on blood pressure measurement conducted by health professionals by asking: ‘When was the last time that your blood pressure was measured by a health professional?’. Five possible responses were given: ‘Within the past twelve months’, ‘One to less than three years’, ‘Three to less than five years’, ‘Five years or more’ and ‘Never’. The answers were used to establish a dichotomous variable for blood pressure checks in the last twelve months (‘yes’/’no’). The questions used for blood cholesterol and blood sugar measurements by medical professionals in the last twelve months used similar wording.

#### Utilisation of medically prescribed drugs

Data on the utilisation of medically prescribed drugs in the two weeks prior to the survey is depicted using the prevalence of current prescription medication. The participants were asked: ‘During the past two weeks, have you used any medicines that were prescribed for you by a doctor? Exclude contraceptive pills or hormones used solely for contraception’. The possible responses were ‘Yes’, ‘No’, ‘Don’t know’ and ‘Prefer not to answer’.

#### Sociodemography

In addition to age, respondents’ gender and education were also taken into account as determinants of health care utilisation. GEDA 2019/2020-EHIS used gender identities to describe gender differences and allowed the respondents to indicate which gender they felt they belonged to. Respondents 18 years and older included 11,959 women and 10,687 men. 62 respondents provided a different gender identity to the one that they were assigned at birth or gave no information at all. These individuals are not included in the gender stratified analyses.

The International Standard Classification of Education (ISCED) was used to classify the information provided by the study participants on education [24]. ISCED takes into account both school and vocational qualifications and is particularly suitable for international comparisons. ISCED categories 0 to 2 were grouped into a low, 3 to 4 into a medium and 5 to 8 into a high education group.

### 2.3 Statistical analyses

The analyses are based on data from 22,646 participants (11,959 women, 10,687 men) aged 18 to 99. Depending on the indicator, participants without information on the variables on which an indicator is based were excluded from the analyses (27 for GPs, 60 for specialists, 11 for psychiatric and psychotherapeutic services, 179 for blood pressure, 684 for blood cholesterol, 1,100 for blood sugar and 3 for medically prescribed drugs). The analysis of utilisation of stool test is based on data from 5,507 participants (3,058 women, 2,449 men). The utilisation of colonoscopy is based on data from 8,408 participants (4,329 women, 4,079 men).

The analyses were carried out using a weighting factor to correct the sample for deviations from the population structure. Design weighting was first carried out for the different selection probabilities (mobile and landline). This was followed by an adjustment to the official population figures based on age, sex, federal state and district type (as of 31 December 2019). Adjustments were also undertaken to ensure the data reflected the education distribution identified by the 2017 microcensus. This was conducted in accordance with ISCED classifications [27].

The analyses were carried out with SAS 9.4. In order to properly account for the weighting when calculating confidence intervals and p-values, all analyses were undertaken using SAS survey procedures. A statistically significant difference between groups is assumed where p-values are less than 0.05.

## 3. Results

### 3.1 General practitioner and specialist utilisation

**Figure 1:**
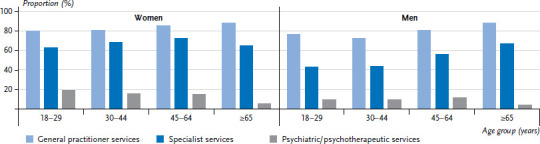
Utilisation of general practitioner, specialist, and psychiatric/psychotherapeutic services in the last twelve months by gender and age (general practitioner services n=11,945 women, n=10,675 men; specialist services n=11,925 women, n=10,663 men; psychiatric/psychotherapeutic services n=11,953 women, n=10,682 men) Source: GEDA 2019/2020-EHIS

84.2% of women and 79.5% of men reported seeing a GP in the last twelve months. Specialist medical services were used less often (women 67.8%, men 53.3%). The utilisation of medical services tends to increase with age while gender differences towards a higher utilisation among women remain (Figure 1 and Annex Table 1). With regard to education, there is a tendency towards a greater utilisation of GP services by people from the lower education group compared with those from the medium and higher education group. The opposite correlation can be found for specialist services, with a more frequent utilisation by people from the higher education group. This relationship is much more pronounced among women than men (Figure 5, Figure 6 and Annex Table 1).

### 3.2 Utilisation of psychiatric and psychotherapeutic services

12.7% of women and 8.9% of men reported that they have used psychotherapeutic and psychiatric services in the past twelve months. The frequency differs between age groups. People aged 65 or over report the lowest utilisation of these services (women 5.3%, men 3.8%). Women between the ages of 18 and 29 do so almost four times as often, at 19.2%. For men, those aged between 45 and 64 most frequently reported having used psychotherapeutic and psychiatric services, at 11.6%, which is about three times the rate identified for men aged 65 or above (Figure 1 and Annex Table 1). Gender differences are also evident when comparing education groups. Although there is no evidence of an educational gradient among women, men in the lower education group seek specialist care for psychological complaints and mental disorders roughly twice as often (13.0%) as men in the higher education group (6.7%) (Figure 5, Figure 6 and Annex Table 1).

### 3.3 Utilisation of stool test and colonoscopy

**Figure 2:**
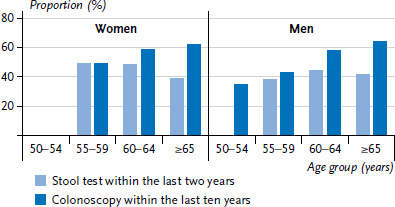
Utilisation of stool test and colonoscopy by gender and age (Stool test n=3,058 women, n=2,449 men; colonoscopy n=4,329 women, n=4,079 men) Source: GEDA 2019/2020-EHIS

In line with the recommendations, around a third of women (34.2%) and around one fifth of men (20.2%) between the ages of 50 and 54 reported having had a stool test in the last twelve months. This difference is significant (data not shown). Considerably more people had a test within the last two years, although hardly any differences were identified in this case between women and men (Figure 2 and Annex Table 2). It is particularly striking that women’s utilisation of stool tests decreases with age: significantly fewer women in other age groups reported a test compared with 55- to 59-year-olds. In contrast, utilisation of stool test tends to increase with age among men. The data show that colonoscopies are reported significantly more often by people aged 60 or above than by younger people.

### 3.4 Blood pressure, blood cholesterol and blood sugar measurement by health professionals

**Figure 3.**
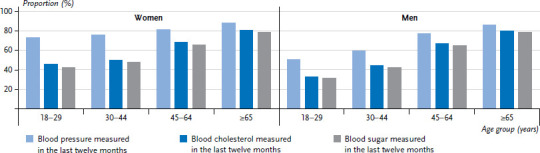
Blood pressure, blood cholesterol, and blood sugar measurement by health professionals in the last twelve months by gender and age (Blood pressure measurement n=11,873 women, n=10,597 men; blood cholesterol n=11,622 women, n=10,341 men; blood sugar n=11,383 women, n=10,168 men) Source: GEDA 2019/2020-EHIS

The percentage of women and men who reported having had a blood pressure check-up undertaken by a health care professional in the past twelve months was 81.0% and 70.7%, respectively. These figures increase significantly with age for both genders. Moreover, they are also significantly higher among women in the 18-to-29 and 30-to-44 age groups than among men of the same age. However, no gender differences were identified among 45- to 64-year-olds or people aged 65 or over (Figure 3). Similar results were obtained for blood cholesterol and blood sugar. For example, 64.7% of women and 59.4% of men report that their blood cholesterol had been checked by health professionals in the last twelve months. 62.3% of women and 57.4% of men report that their blood sugar has been measured by health professionals in the past twelve months. The proportion of people who have had their blood cholesterol and blood sugar levels tested also increases significantly with age. Significant gender differences were only identified between 18- to 29- and 30- to 44-year-olds (Figure 3). With regard to education, no differences were identified for blood pressure and blood cholesterol between people from the lower education group and those from the medium and higher education groups. A smaller proportion of men in the lower education group reported blood sugar check-ups than men in the medium and higher education groups (Figure 6). In women, a clear educational gradient was identified for blood cholesterol and blood sugar measurements, but not for blood pressure. A higher proportion of women in the lower education group reported blood choles terol and blood sugar measurements than women in the medium and higher education groups (Figure 5).

### 3.5 Utilisation of medically prescribed drugs

**Figure 4.**
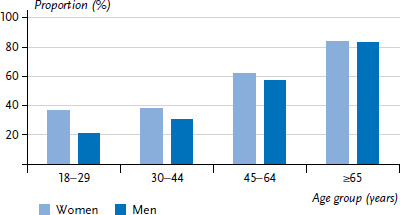
Utilisation of medically prescribed drugs in the last two weeks by gender and age (n=11,958 women, n=10,686 men) Source: GEDA 2019/2020-EHIS

**Figure 5.**
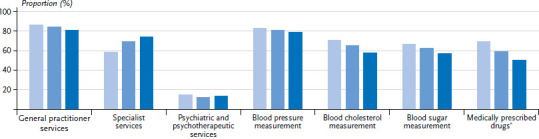
Utilisation of outpatient care services in the last twelve months among women by education level Source: GEDA 2019/2020-EHIS

**Figure 6.**
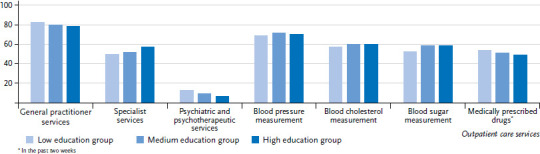
Utilisation of outpatient care services in the last twelve months among men by education level Source: GEDA 2019/2020-EHIS

More than half of the study participants (59.2% of woman, 50.6% of men) reported that they had used medically prescribed drugs in the last two weeks (Figure 4 and Annex Table 1). The prevalence differs significantly during the life course and increases with age: in the youngest age group (18 to 29 years), 36.9% of women and 20.7% of men had used medication prescribed by a doctor in the last two weeks, whereas the prevalence among people aged 65 or above was much higher (83.6% for women and 83.0% for men). Gender differences were recorded in the 18-to-29, 30-to-44 and 45-to-64 age groups, with significantly higher prevalences among women than men. From the age of 65, the prevalences level out. Women from the lower education group (69.3%) have a significantly higher prevalence of medically prescribed drug use than women from the higher education group (50.2%) (Figure 5 and Figure 6). This social gradient was also observed in men, but was not found to be statistically significant.

## 4. Discussion

This article describes key data on the utilisation of outpatient health care services in Germany. In addition to certain preventive services (colorectal cancer screening), focus is placed on the utilisation of general practitioner, specialist and psychiatric/psychotherapeutic services, important medical check-ups and medication. The analyses demonstrate a tendency towards differences by gender in the sense of a higher utilisation of health services by women. Many health services are also used more frequently with increasing age, and educational differences were observed for some of the indicators.

### 4.1 Utilisation of services provided by general practitioners and specialists

Around eight out of ten respondents used general practitioners in the twelve months prior to the survey. Specialist medical services were utilised by around six out of ten respondents within the last year and, thus, somewhat less often. Previous studies have demonstrated a relatively high utilisation of outpatient medical services in Germany [2, 26]. An initial analysis of the GEDA 2019/2020-EHIS data over time in the initial phase of the COVID-19 pandemic showed that the utilisation of general and specialist medical services fell briefly, albeit significantly, in 2020 when containment measures were in place [27]. This study presumably therefore slightly underestimates the utilisation of outpatient medical services in terms of the average level over the entire study period. A higher level of utilisation with increasing age as a result of increasing morbidity is also well documented in the literature on factors influencing the utilisation of many health services; as is the generally higher level among women [11, 26]. Gender differences are often explained in terms of women having a higher physical sensitivity and a greater willingness to accept help and to make greater use of preventive services. Men are viewed as more inclined to take advantage of medical services only after diseases already have appeared [26]. This also explains the trend towards a decrease in gender differences with increasing age as more treatment is needed in older age due to rising morbidity. Socioeconomic differences in health care can already be found in childhood [28]. In addition, the tendency towards a higher utilisation of general medical services with decreasing socioeconomic status is also well-known. In the present analysis this was operationalised using the respondents’ educational level. The findings go hand in hand with the tendency of people with a higher socioeconomic status to make greater use of specialist medical services [29, 30]. These socioeconomic differences are partly explained by the fact that in Germany, people with lower socioeconomic status often use general practitioners as gatekeepers (i.e. people who guide them through the health system) and only utilise specialist services on their advice [31]. The differences between health systems in Europe mean that Europe-wide comparisons are only possible to a limited extent. Data from EHIS Wave 2 for 2014 show that both the outpatient utilisation of GP and specialist medical services are relatively high in Germany compared with other EU member states; the utilisation of psychiatric and psychotherapeutic services is also above the EU average [32].

### 4.2 Utilisation of psychiatric and psychotherapeutic services

12.8% of women and 8.9% of men reported having had psychotherapeutic or psychiatric counselling or treatment in the past twelve months. Data from BARMER health insurance for 2018 also show that a comparable proportion of the population was treated by psychological psychotherapists (3.1%) and psychiatrists and neurologists (10.9%) [33]. Assuming that 27.8% of the population are affected by a mental disorder at least once a year [34, 35], the utilisation of specialist services can be described as low. Given the fact that almost three quarters of patients with a documented diagnosis of a mental disorder only received treatment from a GP or specialist in somatic medicine [36], a treatment gap in the provision of care for mental health is discussed.

Compared with the results of GEDA 2014/2015-EHIS, the analyses set out here identified a slight increase in the utilisation of psychotherapeutic and psychiatric services over time (women 11.3%, men 8.1%). This particularly applies to women in young adulthood (18 to 29 years of age) as the figure for this group increased by 8.7 percentage points [13]. For psychotherapeutic services, this peak in the age distribution, which has become increasingly pronounced over the last few years, is also found in health insurance data [33]. Furthermore, these data demonstrate that the currently still low level of utilisation by people aged 65 or above (see also [37, 38]) has increased in recent years. Taking into account that, for example, the frequency of depression diagnoses increases with age, care provision in this context becomes increasingly needs-based over time [39]. Apart from this, the age distribution of the utilisation of psychotherapeutic services identified from data from statutory health insurers [33] differs significantly from the findings presented here, because our study includes psychiatric (and psychological) care, which are known to have different age distributions [40].

The finding that women seek psychiatric and psychotherapeutic help more often than men is confirmed by the literature [41]. Furthermore, our results demonstrate that the educational differences in the utilisation of services also vary between the genders. Men in the low education group have a more frequent rate of utilisation, reflecting that mental distress and disorders occur more frequently in people with lower income and educational and professional status [34]. Although this difference was also expected among women, no evidence was found to support it in the data used here. This could be due to the fact that social inequality in mental disorders is more pronounced in men than in women [42]. In addition, when collecting data on the utilisation of services, occupational groups were considered together, although they would presumably have to be looked at separately in this regard, too. Numerous signs indicate that especially persons with higher levels of education have easier access to outpatient psychotherapeutic services in particular, and that these seem to differ from psychiatric and possibly psychological services [33, 38, 43, 44]. Since women make more use of psychotherapy than men, this may lead to the appearance that the services are being utilised equally by women of all education groups – which is unjustified because of the social gradient of morbidity.

### 4.3 Utilisation of stool test and colonoscopy

The analyses of the available data show that a relatively large number of people over the age of 50 (around 40%) report having had a stool test within the last two years. The figures for a colonoscopy within the last ten years are even higher, at more than 50%. Both tests can be used preventively as part of colorectal cancer screening but also to determine the cause of symptoms. As no data was collected as part of GEDA 2019/2020-EHIS on the reasons for conducting the tests, the proportion used for screening remains unclear. However, figures can also be gained from claims data from statutory health insurers [8]. GEDA 2019/2020-EHIS identified a significantly higher rate of stool tests than found among claims data, which indicates that stool tests are often not carried out or billed as screening measures, but rather to determine the cause of symptoms. In the case of colonoscopies, the figures identified from self-reported data are significantly higher than those attained from claims data. Other studies have also identified comparatively high numbers of colonoscopies from self-reported data [45]. A study based on claims data from AOK Hessen found the ratio of preventive to curative colonoscopies to be about 1:2 among 50- to 79-year-olds and even 1:4 among people aged 80 or above [46]. These results are therefore of a similar magnitude to those from GEDA 2019/2020-EHIS. A comparison with the data from GEDA 2014/2015-EHIS once again demonstrates very little change in the figures from self-reported data [9].

International comparisons of stool tests and colonoscopies as part of colorectal cancer screening need to be regarded with caution because of the differences between screening programs in different countries [47]. A European-wide comparison of data from EHIS Wave 2 for 2014, however, ranked Germany third after France and Slovenia in terms of utilisation of a stool test among 50- to 74-year-olds within the last two years. The European average among the then 28 member states in this age group was 31.3% [48]. The European average for colonoscopy utilisation among 55- to 64-year-olds was 25.7%. In addition to Germany, Austria and Luxembourg also reported figures over 50% [49].

Differences by gender are only apparent with regard to the stool test. Since gynaecologists can also offer this test, women may have more of an opportunity to be tested, for example during cervical cancer and breast cancer screening. This assumption ties in with the fact that women take stool tests less often as they get older. The use of the Pap smear for cervical cancer screening also decreases significantly with age [9]. This suggests that older women generally no longer regularly make use of gynaecological services [50].

In terms of colonoscopy utilisation, a significant increase with age was identified both among women and men. Colonoscopies, therefore, tend not to be undertaken at the beginning of the period in which most people are eligible. This could be due to the fact that a colonoscopy is a relatively complex and invasive procedure and therefore requires longer-term planning. In addition, the increasing utilisation of medical services by men with age could explain the increased utilisation of colonoscopies as well as rebalance the earlier differences identified between women and men [2].

### 4.4 Blood pressure, blood cholesterol and blood sugar measurement by health professionals

High blood pressure, blood cholesterol and blood sugar levels are major risk factors in the development of cardiovascular disease and diabetes. Regular tests can determine elevated and borderline elevated levels in people without known diseases (hypertension, hyperlipidaemia, diabetes). People with known diseases require regular monitoring of blood pressure, blood cholesterol and blood sugar levels for drug treatment, and this may even be set out in therapy guidelines. Therefore, medical services (monitoring of blood pressure, blood cholesterol and sugar) are presumably more likely to be utilised by patients with these known diseases. For example, the proportion of people with known diabetes who have had their blood sugar tested by a health professional in the past twelve months is 96.3%, compared with 56.0% for people without known diabetes (data not shown).

In Germany, people with statutory health insurance aged 35 or over are entitled to a medical health check-up, and an integral part of this check-up is a blood test for sugar and cholesterol [51]. Since April 2019, this health check-up has been offered every three years to people aged 35 or above and once to people aged between 18 and 34 [51]. The analyses presented here show that the majority of study participants aged 18 or over had had their blood pressure, blood cholesterol and blood sugar checked by health professionals in the past twelve months. These results reflect similar figures for health check-ups in Germany. Claims data from statutory health insurers show that around half of the population with statutory health insurance aged 35 or above had a health check-up in 2017/2018 [52]. Since blood pressure measurement and diagnostic blood tests are routine aspects of health care services provided by GPs and specialists, and because the majority of women and men have received health care from a GP or specialist in the last twelve months, these figures are consistent with those on the frequency of blood pressure, blood cholesterol and blood sugar check-ups being carried out in the past twelve months.

Although the prevalence of each of the three tests increases with age, a significant difference between women and men is also identifiable [52]. Women have a higher prevalence for blood pressure testing by a health care professional. This difference was also observed from the data collected by GEDA 2014/2015-EHIS (women 83.4% vs men 72.5%). Nevertheless, those figures are for the population aged 15 and over [53]. Gender differences in awareness, management and control of hypertension are also known, but the German Health Interview and Examination Survey for Adults (DEGS1, 2008–2011) conducted by the RKI found no differences between women and men with known hypertension in terms of their uptake of blood pressure monitoring by medical professionals [54].

On the international level, the 2017 Swiss Health Survey collected data on blood pressure, blood cholesterol and blood sugar tests from the majority of the population aged 15 or over. The study found that in 2017, the blood pressure of 76.4% (women 81.7%, men 70.9%), the cholesterol level of 45.8% (women 46.7%, men 44.8%) and the blood sugar level of 51.5% (women 54.1%, men 48.8%) of the Swiss population had been measured within the last twelve months. The proportion of female participants was higher [55]. According to data from EHIS Wave 2 (2014), 51.6% of the EU population aged 15 or over reported that their blood cholesterol level had been measured within the last year; 51.0% reported a blood sugar test [48].

### 4.5 Utilisation of medically prescribed drugs

The utilisation of medically prescribed drugs in the two weeks prior to the survey shows the prevalence of current, medically prescribed drug use among adults in Germany. The prevalence described in this study is similar to the prevalence calculated in 2014/2015 (55.5% vs 55.1%) [6]. Significant gender differences in the utilisation of medically prescribed drugs were recorded in both GEDA 2014/2015-EHIS and GEDA 2019/2020-EHIS, especially in younger age groups (under 64 years of age), with higher prevalence among women than men. Prevalences between women and men are similar as of the age of 65. The use of prescribed medication increases with age, and this can be attributed to the increasing prevalence of chronic diseases with age [6, 56]. EHIS Wave 2 found the average utilisation of medically prescribed drugs in people aged 15 and over in the EU to be 48.6% in 2014 [57].

### 4.6 Strengths and Limitations

The data used for GEDA 2019/2020-EHIS is self-reported and may be affected by limitations such as recall bias. There is some evidence that the actual number of physician visits is often underestimated, particularly by older people [58]. However, this mainly applies to data collections on the number of physician visits and less to the question as to whether physicians were consulted at all. Recall bias is more likely for periods lasting longer than twelve months [59]. In addition, telephone interviews are also known to be more susceptible to socially desirable responses than face-to-face interviews, and this can especially be the case when using preventive services such as cancer screening [60].

As response rates for telephone surveys are generally lower than for face-to-face interviews, telephone-based surveys may be at a greater risk of non-response bias. However, a lower response rate does not automatically mean that the results are more strongly biased [61]. Nevertheless, there is still a possibility of selective non-participation (selection bias) [16]. People who take part in health surveys can be assumed to have a greater awareness about health and, therefore, their utilisation of outpatient health services may differ from that of the general population. Furthermore, certain population groups may be underrepresented, such as migrants who lack sufficient knowledge of German to answer the survey questions. One of the strengths of the GEDA study is that selection effects were taken into account by weighting. As such, results from the study are general-isable for Germany. In contrast to claims data, which are often only meaningful for certain groups of insurants and are limited to the information required for accounting purposes and details of prescribed medication [15, 16], survey data can provide information about people with all kinds of health insurance (including private insurance) and on the medicines that were actually taken [16].

The data collection period for GEDA 2019/2020-EHIS overlapped with the COVID-19 pandemic. The results set out here are based on the assumption that the sample showed no systematic bias due to the measures taken to contain the COVID-19 pandemic. Although initial analyses have indeed identified no systematic selection bias between the subsamples from the comparison periods 2019 and 2020, a change in willingness to participate and an impact on the results cannot be completely ruled out. The use of short-time working and the expansion of flexible work from home may, for example, have made it easier (or more difficult) to reach certain population groups by telephone.

The analyses set out here are based on questions from the EHIS questionnaire, which was integrated into the GEDA study. The joint query on the occupational groups of psychiatric, psychotherapeutic and psychological treatment providers, as specified by the EHIS, means that it is impossible to differentiate between their respective specific utilisation. This makes it difficult to compare results with those from other data sources and, for example, masks educational differences. One advantage of this method, however, is that data on the utilisation of specialised care services for mental health complaints and disorders are collected as a whole; these data can then be compared with the frequency of these complaints in the population so as to identify discrepancies in care provision and gaps in utilisation.

### 4.7 Conclusion

This article describes the utilisation of various outpatient services using current representative population-based data. The vast majority of the population utilises outpatient health care services at least once a year.

Only a more differentiated view calling for in-depth analyses reveals that in part the utilisation and its development over time has varied greatly in recent years for different age and population groups. Different utilisation rates among different population groups can be attributed to various causes: in addition to particular medical needs, this includes patient preferences, such as for visiting a GP or a specialist, the availability of care, information about available health services, and access barriers. When comparing the utilisation of specialist services in general to psychiatric/psychotherapeutic utilisation, a deviating educational gradient is noticeable, especially among men. This may indicate barriers to care, varying in terms of specialist groups and the health conditions in question. Early detection and treatment of colorectal cancer are among the measures that have been shown to reduce mortality at the population level. In order to break down existing barriers to utilisation, the specific needs of those eligible, but also their personal attitudes and beliefs, should be given greater consideration. If these services are to become more accessible, research is needed into possible barriers to utilisation, especially in the case of younger people. In principle, qualitative research designs could be used to study non-utilisation of outpatient health services.

Overall, the data from GEDA 2019/2020-EHIS are an important source of information for health services research.

Together with data from service providers and structural data on health care provision, they provide a basis with which to undertake comprehensive descriptions of health care provision in Germany. European comparisons can currently only be made to a limited extent, but this will change in the future when all European data from this wave of the EHIS wave become available.

## Key statements

- Around 80% of the population aged 18 or above used general practitioner services at least once a year. Around 60% seeked specialist medical care.
- Psychiatric and psychotherapeutic services are most commonly utilised by women aged between 18 and 29.
- The utilisation of colonoscopies, which rises with age, is not associated with education level or gender.
- The utilisation of blood pressure, blood cholesterol and blood sugar check-ups increases with age and is more common among women than men, and particularly among 18- to 44-year-old women.
- The utilisation of medically prescribed drugs is higher among women, the elderly and people in the lower education group.

## Appendix

**Annex Table 1:** Utilisation of outpatient care services by gender, age and education level Source: GEDA 2019/2020-EHIS

	Use of general practitioner services in the last twelve months (n=11,945 women, n=10,675 men)		Use of specialist services in the last twelve months (n=11,925 women, n=10,663 men)		Use of psychiatric/psychotherapeutic services in the last twelve months (n=11,953 women, n=10,682 men)		Blood pressure measurement in the last twelve months (n=11,873 women, n=10,597 men)		Blood cholesterol measurement in the last twelve months (n=11622 women, n=10,341 men)		Blood sugar measurement in the last twelve months (n=11,383 women, n=10,168 men)		Utilisation of medically prescribed drugs in the last two weeks (n=11,958 women, n=10,686 men)	
	%	(95% CI)	%	(95% CI)	%	(95% CI)	%	(95% CI)	%	(95% CI)	%	(95% CI)	%	(95% CI)
**Total**														
Women	84.2	(83.2–85.2)	67.8	(66.5–69.1)	12.7	(11.8–13.7)	81.0	(79.9–82.1)	64.7	(63.3–66.0)	62.3	(60.9–63.7)	59.2	(57.9–60.6)
Men	79.5	(78.2–80.7)	53.3	(51.8–54.7)	8.9	(8.0–9.8)	70.7	(69.3–72.0)	59.4	(57.9–60.8)	57.4	(55.9–58.9)	50.6	(49.1–52.0)
**Life stage (age group)**														
**Women**														
18–29 years	79.6	(76.1–82.7)	62.8	(58.6–66.9)	19.2	(15.7–23.1)	72.9	(69.0–76.5)	46.2	(41.8–50.7)	42.7	(38.3–47.2)	36.9	(32.8–41.2)
30–44 years	80.6	(78.0–82.9)	68.1	(64.9–71.1)	15.2	(13.0–17.6)	76.2	(73.5–78.8)	50.3	(47.0–53.5)	48.1	(44.8–51.3)	38.0	(35.0–41.1)
45–64 years	85.3	(83.8–86.7)	72.3	(70.3–74.1)	14.5	(13.0–16.1)	81.3	(79.6–83.0)	68.2	(66.3–70.1)	65.8	(63.9–67.8)	62.2	(60.2–64.1)
≥65 years	88.2	(86.5–89.6)	64.9	(62.5–67.3)	5.3	(4.4–6.5)	88.4	(86.9–89.8)	80.5	(78.6–82.2)	78.4	(764–80.3)	83.6	(81.8–85.1)
**Men**														
18–29 years	76.4	(73.1–794)	42.6	(39.0–464)	8.9	(7.0–11.6)	50.6	(46.8–544)	33.1	(29.6–36.8)	31.5	(28.0–35.3)	20.7	(17.8–23.9)
30–44 years	72.0	(68.9–74.9)	43.4	(40.2–46.6)	9.5	(7.7–11.7)	59.6	(56.3–62.8)	44.5	(41.2–47.9)	42.5	(39.2–45.9)	30.8	(27.8–34.0)
45–64 years	80.4	(78.5–82.1)	56.2	(53.9–58.5)	11.6	(10.0–13.3)	77.1	(75.2–79.0)	67.2	(64.9–694)	64.7	(624–67.0)	57.1	(54.7–59.3)
≥65 years	87.9	(85.9–89.7)	66.6	(63.9–69.1)	3.8	(3.0–4.9)	86.5	(844–88.3)	80.3	(77.9–82.5)	78.6	(76.1–80.9)	83.0	(80.8–85.0)
**Education group**														
**Women**														
Low	86.3	(83.2–88.9)	58.2	(54.2–62.2)	14.8	(12.1–17.9)	83.0	(79.7–85.8)	70.3	(66.4–74.0)	66.5	(62.5–70.3)	69.3	(65.4–72.9)
Medium	84.6	(83.3–85.9)	69.1	(67.5–70.7)	11.9	(10.7–13.2)	81.2	(79.7–82.5)	65.1	(63.4–66.8)	62.6	(60.9–64.3)	58.9	(57.2–60.6)
High	80.9	(79.3–82.4)	74.2	(72.4–75.8)	13.1	(11.7–14.5)	79.0	(77.4–80.5)	57.8	(55.9–59.7)	57.3	(55.3–59.2)	50.2	(48.3–52.1)
**Men**														
Low	82.6	(78.1–86.4)	49.9	(44.6–55.3)	13.0	(9.6–17.2)	68.5	(63.3–73.3)	57.0	(51.4–62.5)	52.0	(46.4–57.5)	54.0	(48.7–59.3)
Medium	78.2	(77.6–81.0)	51.9	(49.8–53.9)	9.0	(7.9–10.3)	71.6	(69.6–73.4)	59.7	(57.6–61.8)	58.2	(56.0–60.2)	50.9	(48.9–53.0)
High	78.2	(76.8–79.6)	57.4	(55.8–59.0)	6.7	(5.9–7.5)	70.1	(68.5–71.6)	59.9	(58.3–61.5)	58.5	(56.8–60.1)	48.6	(47.0–50.2)

CI=Confidence interval

**Annex Table 2:** Utilisation of stool tests and colonoscopies by gender, age and education level Source: GEDA 2019/2020-EHIS

	Stool test within the last two years (n=3,058 women, n=2,449 men)		Colonoscopy within the last ten years (n=4,329 women, n=4,079 men)	
	%	(95% CI)	%	(95% CI)
**Total**				
Women	42.5	(40.7–44.3)	58.7	(56.9–60.5)
Men	41.5	(39.5–43.5)	53.4	(51.5–55.2)
**Life stage (age group)**				
**Women**				
50–54 years	–	–	–	–
55–59 years	49.3	(45.5–53.0)	49.5	(45.8–53.2)
60–64 years	48.2	(44.2–52.3)	58.6	(54.6–62.5)
≥65 years	38.6	(36.3–40.9)	61.8	(59.4–64.1)
**Men**				
50–54 years	–	–	–	–
55–59 years	38.1	(33.9–42.6)	42.9	(38.6–47.3)
60–64 years	44.2	(40.0–48.5)	58.2	(53.8–62.4)
≥65 years	41.7	(39.1–44.3)	63.9	(61.2–66.6)
**Education group**				
**Women**				
Low	38.2	(33.6–43.0)	57.9	(53.1–62.6)
Medium	43.6	(41.6–45.7)	58.8	(56.8–60.8)
High	45.9	(43.7–48.1)	59.5	(57.3–61.7)
**Men**				
Low	35.8	(27.9–44.7)	49.5	(41.4–57.6)
Medium	42.0	(39.2–44.9)	51.9	(49.2–54.5)
High	42.1	(40.2–44.1)	57.1	(55.2–58.9)

CI=Confidence interval
